# Read-through transcription of tRNA underlies the cell cycle-dependent dissociation of IHF from the DnaA-inactivating sequence *datA*

**DOI:** 10.3389/fmicb.2024.1360108

**Published:** 2024-02-28

**Authors:** Kazutoshi Kasho, Ryuji Sakai, Kosuke Ito, Wataru Nakagaki, Rion Satomura, Takafumi Jinnouchi, Shogo Ozaki, Tsutomu Katayama

**Affiliations:** Department of Molecular Biology, Graduate School of Pharmaceutical Sciences, Kyushu University, Fukuoka, Japan

**Keywords:** *E. coli*, DnaA, *datA*, IHF, tRNA transcription, cell cycle

## Abstract

Timely initiation of chromosomal DNA replication in *Escherichia coli* is achieved by cell cycle-coordinated regulation of the replication origin, *oriC*, and the replication initiator, ATP-DnaA. Cellular levels of ATP-DnaA increase and peak at the time for initiation at *oriC*, after which hydrolysis of DnaA-bound ATP causes those to fall, yielding initiation-inactive ADP-DnaA. This hydrolysis is facilitated by the chromosomal locus *datA* located downstream of the tRNA-Gly (*glyV-X-Y*) operon, which possesses a cluster of DnaA-binding sequences and a single binding site (IBS) for the DNA bending protein IHF (integration host factor). While IHF binding activates the *datA* function and is regulated to occur specifically at post-initiation time, the underlying regulatory mechanisms remain obscure. Here, we demonstrate that *datA*-IHF binding at pre-initiation time is down-regulated depending on the read-through transcription of *datA* IBS initiated at the *glyV-X-Y* promoter. During the cell cycle, the level of read-through transcription, but not promoter activity, fluctuated in a manner inversely related to *datA*-IHF binding. Transcription from the *glyV-X-Y* promoter was predominantly interrupted at *datA* IBS by IHF binding. The terminator/attenuator sequence of the *glyV-X-Y* operon, as well as DnaA binding within *datA* overall, contributed to attenuation of transcription upstream of *datA* IBS, preserving the timely fluctuation of read-through transcription. These findings provide a mechanistic insight of tRNA transcription-dependent *datA*-IHF regulation, in which an unidentified factor is additionally required for the timely *datA*-IHF dissociation, and support the significance of *datA* for controlling the cell cycle progression as a connecting hub of tRNA production and replication initiation.

## Introduction

**FIGURE 1:**
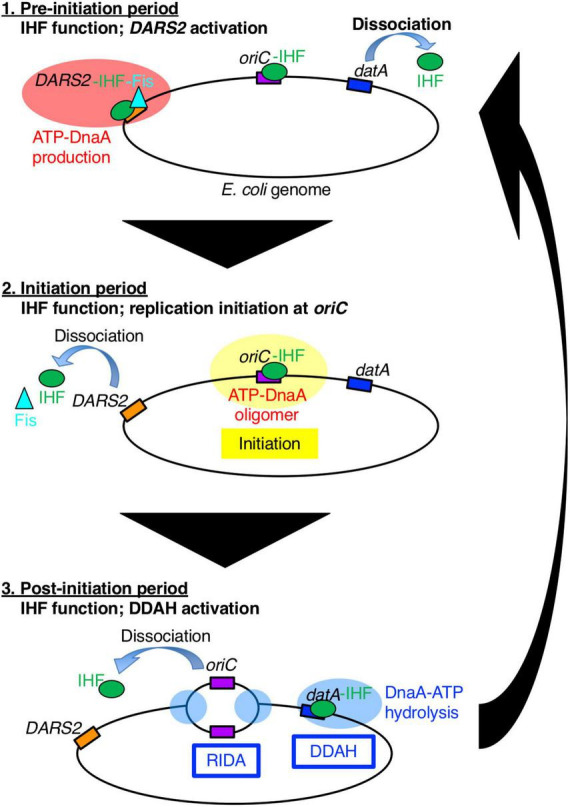
A schematic view of the timely binding/dissociation of IHF and Fis at *oriC*, *datA*, and *DARS2* during the cell cycle. At the pre-initiation stage, during which the ADP-DnaA level is high, the *DARS2*–IHF–Fis complex increases the level of ATP-DnaA. Concurrently, IHF dissociates from *datA*. Next, at the initiation stage, the elevated ATP-DnaA level results in initiation of replication at the IHF-bound *oriC* and Fis dissociation from *DARS2*. Concurrently, IHF dissociates from *DARS2* via an unknown mechanism. After initiation, IHF temporarily binds to *datA* and activates DDAH to inactivate DnaA. For simplicity, IHF and Fis proteins are drawn as a green circle and a light blue triangle, respectively, and DnaA protein is omitted.

The *E. coli* chromosome is a circular double-strand 4.6 Mb DNA molecule that forms a compact structure, the nucleoid, condensed by binding of various nucleoid-associated proteins (NAPs) and a DNA superstructure, such as supercoiled DNA (Dillon and Dorman, 2010; Hołówka and Zakrzewska-Czerwińska, 2020; Molan and Žgur Bertok, 2022). The *E. coli* chromosome has a single replication origin, *oriC* (located at 84.6 min of the genome map), and initiation of replication at *oriC* is regulated precisely during the cell cycle so that, even in rapidly growing cells, initiation occurs simultaneously at multiple sister *oriCs* (Frimodt-Møller et al., 2017; Hansen and Atlung, 2018; Grimwade and Leonard, 2021; Kasho et al., 2023). In *E. coli*, the initiation reactions of DNA replication occur in a higher-order complex that includes *oriC*, the initiator protein DnaA, and a NAP IHF (integration host factor). The *oriC* region comprises a duplex-unwinding element (DUE) and a flanking DnaA oligomerization region (DOR), which contains a cluster of DnaA-binding sites (DnaA boxes). ATP-bound DnaA, but not ADP-bound DnaA, forms specific oligomers with the DOR that promote DUE unwinding in concert with IHF or its homologue HU (Cassler et al., 1995; Shimizu et al., 2016; Sakiyama et al., 2017; Kasho et al., 2021; Yoshida et al., 2023). IHF specifically binds to a consensus sequence (TAAnnnnTTGATW, where W is A or T, and n is any nucleotides) at a DUE-proximal site within the DOR and induces sharp DNA bending (120°–180°) (Figure 1; Aeling et al., 2006). This promotes specific binding of a DOR-bound DnaA oligomer to the single-stranded (ss) DUE, thus stabilizing the unwound state of DUE. The stably unwound DUE causes subsequent loading of the replicative DNA helicase, DnaB, onto the ssDUE (Hayashi et al., 2020), thereby initiating DNA synthesis by the DnaG primase and the DNA polymerase III holoenzyme.

*E. coli* DnaA is composed of four distinct domains, whose basic features are conserved among other bacteria (Katayama et al., 2017). The N-terminal domain I is involved in interactions with DnaB and DiaA, a stimulatory factor for ATP-DnaA assembly on *oriC* (Sutton et al., 1998; Keyamura et al., 2009; Zawilak-Pawlik et al., 2017). Domain II is a flexible linker (Nozaki and Ogawa, 2008). Domain III contains AAA+ motifs involved in ATP/ADP binding (Kawakami et al., 2006), ATP hydrolysis (Nishida et al., 2002), and domain III–domain III interactions (Kawakami et al., 2005; Erzberger et al., 2006; Noguchi et al., 2015), in addition to a specific motif for ssDUE binding (Ozaki et al., 2008; Duderstadt et al., 2011; Yoshida et al., 2023). Domain IV binds specifically to the 9-mer DnaA box consensus sequence, TTAWnCACA (where W is A and T) (Fujikawa et al., 2003).

Integration host factor exists as a heterodimer of α- and β-subunits and is abundant in *E. coli* cells (approximately 5,000 molecules per cell at log phase and 10,000 molecules per cell at stationary phase) (Ali Azam et al., 1999). In addition to replication initiation, IHF regulates chromosome conformation and transcription of hundreds of genes by inducing DNA bending, or modulating DNA supercoiling (Prieto et al., 2012). To achieve precise regulation of replication initiation timing, IHF plays essential roles not only in the timely activation of *oriC*, but also in the timely regulation of DnaA activity via the chromosomal loci *DARS2* and *datA* (Figure 1; Kasho and Katayama, 2013; Kasho et al., 2014). *DARS2* catalytically stimulate the release of ADP from DnaA to produce ATP-DnaA (Fujimitsu et al., 2009; Sugiyama et al., 2019). *DARS2* contains specific binding sites for IHF and another NAP, Fis (factor for inversion stimulation), and is activated during the cell cycle in a manner dependent on the temporal binding of IHF and Fis (Kasho et al., 2014). A recent study revealed that *DARS2* is regulated by a negative feedback mechanism (Figure 1; Miyoshi et al., 2021): at the pre-initiation stage, the increased ATP-DnaA molecules form oligomers at the Fis binding site, competitively dissociate Fis. This simple negative feedback regulation of *DARS2* is fundamental for the timely repression of *DARS2* activity; however, it is currently unclear how IHF binding/dissociation at the *DARS2* locus is regulated during the cell cycle.

**FIGURE 2:**
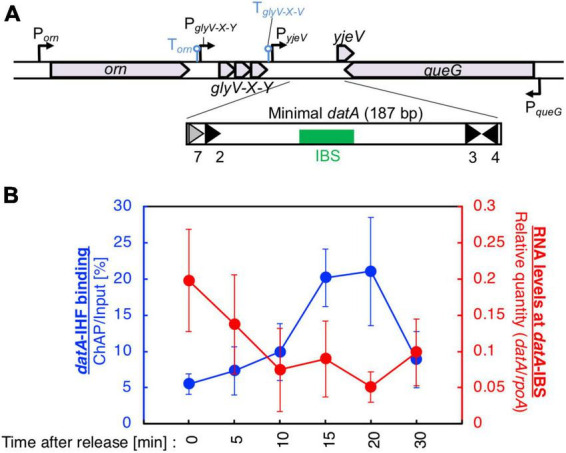
Cell cycle-dependent oscillation of *datA*-IHF binding and *datA* transcription level. **(A)** Functional structures of *datA* and surrounding regions are shown. The location and direction of transcriptional promoters and genes around the chromosomal *datA* and *DARS2* loci are indicated by thin arrows and pentagons. DnaA boxes 3–4 and 7 within the minimal *datA* are indicated by gray triangles (low-affinity boxes with 2–3 mismatches) and black triangles (high affinity boxes with 0–1 mismatch). IBS is indicated by a green box. **(B)** Cell cycle-coordinated correlation between *datA*-IHF binding (blue line) and *datA* transcription (red line). *datA*-IHF binding was analyzed by ChAP-qPCR. SH022 (*dnaC2 ihfA-cHis12*) cells growing in LB medium at 30°C were transferred to 37°C and incubated for 80 min. The cells were then transferred to 30°C (Time 0) and further incubated for 5–30 min at 30°C. The relative levels of *datA* before and after Ni-affinity purification were determined using real-time qPCR, and yield was calculated (expressed as %). Cell cycle-coordinated oscillation of the RNA level of *datA* IBS was analyzed by RT-qPCR. SH022 cells were cultured as described above before the total RNA was extracted. RNA levels of *datA* IBS relative to those of the *rpoA* gene were determined using RT-qPCR. Data represent means (± S.D.) of at least four independent experiments. Based on the Student’s t-test, the 0 min vs. 20 min difference (*p* < 0.02) of RT-qPCR is statistically significant, and 0 min vs. 15 min (*p* < 0.01), 0 min vs. 20 min (*p* < 0.05), and 15 min vs. 30 min (*p* < 0.05) differences of IHF ChAP are statistically significant.

The main system for inactivation of DnaA is called RIDA (regulatory inactivation of DnaA), in which DnaA-bound ATP is catalytically hydrolyzed by the complex of the AAA+ Hda protein and the DNA-loaded form of the clamp (β subunit) of the DNA polymerase III holoenzyme in a manner coupled with DNA replication (Katayama et al., 2010, 2017). However, for strict regulation that represses untimely initiation, the specific chromosomal locus *datA* (located at 94.7 min) is required: *datA* efficiently inactivates ATP-DnaA by DnaA-ATP hydrolysis in a manner dependent on IHF and independent of RIDA. This regulatory system is termed DDAH (*datA*-dependent DnaA-ATP hydrolysis) (Figure 1; Kasho and Katayama, 2013). Deletion of *datA* causes untimely initiations in growing cells (Nozaki et al., 2009; Kasho et al., 2017). The minimal *datA* region contains a single IBS and four DnaA boxes, of which three (DnaA boxes 2, 3, and 7) are essential and one (DnaA box 4) is stimulatory for *datA* function (Figure 2A; Kasho et al., 2017). The *datA*-IHF complex interacts with ATP-DnaA, catalytically promoting the hydrolysis of DnaA-bound ATP. Timely regulation of DDAH depends on the temporal binding of IHF to *datA* (Kasho and Katayama, 2013). IHF is dissociated from *datA* ahead of initiation and binds to *datA* only after initiation, ensuring the timely activation of DDAH. However, the mechanism for ensuring the timing of *datA*-IHF binding/dissociation remains unknown.

Previous studies have presented three clues relating to regulatory mechanisms for *datA* function. (1) Chromosomal positional effects of *datA*, i.e., when the locus of *datA* is moved to a position distant from *oriC*, *datA* function is moderately inhibited, resulting in precocious initiations (Kitagawa et al., 1998; Frimodt-Møller et al., 2015). The mechanisms responsible for this effect remain to be investigated. Local structure of the chromosome, transcriptional activities surrounding *datA* etc. in addition to distance itself from *oriC* are suggested to possibly influence *datA* function as described below. (2) DNA supercoiling-dependent stimulation of DDAH, i.e., negative DNA supercoiling stabilizes *datA-*IHF binding *in vitro* and stimulates DDAH activity (Kasho et al., 2017). In support of this hypothesis, inhibition of negative DNA supercoiling by the DNA gyrase inhibitor novobiocin decreases the ability of *datA* to repress replication initiation *in vivo*. IHF preferentially binds to curved DNA rather than to straight-shaped DNA (Watson et al., 2022). (3) Transcription of *datA*, i.e., the minimal region of *datA* required for full DDAH activity is located at the intergenic region downstream of the tRNA-Gly (*glyV-X-Y*) operon and *queG* gene (Figure 2; Kitagawa et al., 1996; Kasho and Katayama, 2013), which enables a hypothesis that *datA*-IHF binding/dissociation is regulated by transcription of the *datA* locus. The transcriptional promoter of the putative *yjeV* gene (P_*yjeV*_) partly overlaps with *datA* DnaA box 7, which is essential for DDAH activity. Consistent with this hypothesis, the transcription inhibitor rifampicin inhibits timely binding and dissociation of *datA*-IHF complexes, and *datA* activity is inhibited by the translocation of *datA* to a highly transcribed position (Kasho and Katayama, 2013; Frimodt-Møller et al., 2015). However, it is currently unclear which mechanism is predominant for regulating the *datA*-IHF binding/dissociation.

In this study, we addressed the requirement of transcription of the *datA* region for timely regulation of IHF binding/dissociation. We found that *datA*-IHF binding is down-regulated depending on the read-through transcription of *datA* IBS initiated at the promoter of tRNA-Gly operon, which ensures timely *datA*-IHF binding. Cell cycle analyses suggest that the levels of read-through transcripts of *datA* IBS and *datA*-IHF binding were inversely correlated. We next addressed the mechanism of cell cycle-coordinated switching between read-through transcription and premature transcription termination. Multifaceted analysis using various mutants suggested that transcription initiation from the *glyV-X-Y* promoter is constant and premature transcription termination at the *datA* IBS region occurs in a temporal manner. Timely transcription termination predominantly requires IHF binding to *datA* IBS. Also, the terminator/attenuator sequence of *glyV-X-Y* operon and DnaA binding at the *datA* DnaA box 2 contribute to controlling the basal level of transcription termination. Based on these findings, we propose a model and significance of tRNA transcription-dependent regulation for timely *datA*-IHF binding/dissociation for ensuring precise timing of replication initiation.

## Materials and methods

### Bacterial strains and cultures

The *E. coli* strains used in this study are listed in Table 1. The λRED system was used to construct chromosomal mutations (Datsenko and Wanner, 2000). Briefly, PCR fragments including mutated *gly-V-X-Y* operon or *datA* and *frt-kan* gene was transformed into the strain bearing pKD46 (λ RED expression plasmid). To verify the presence of specific mutations in each strain constructed in this study, the chromosomal DNA regions of interest were PCR amplified and analyzed by Sanger sequencing. The detail of λRED system was described below. All bacterial strains were grown in LB medium or M9 medium supplemented with 0.2% casamino acids, 5 μg/mL thiamine, and 0.2% glucose.

**TABLE 1:** List of *E. coli* strains used in this study.

Strain	Genotype	Source
MG1655	WT	Laboratory stock
DH5α	s*upE44*Δ*lacU169 (φ80lacZ*Δ*M15) hsdR17 recA1 endA1 gyrA96 thi-1 relA1*	Laboratory stock
KYA018	MG1655 *dnaC2*	Kasho and Katayama (2013)
SH022	MG1655 *ihfA-*cHis12 *dnaC2*	Kasho et al. (2014)
ITK9c	SH022 Δ*glyV-X-Y::kan*	This work
ITK9	MG1655 Δ*glyV-X-Y::kan*	This work
ITK3c	SH022 *nrr*::*frt-kan*	This work
ITK4c	SH022 ΔP_orn_::GAATTC *nnr*::*frt-kan*	This work
ITK5c	SH022 ΔP_glyVXY_::GAATTC *nrr*::*frt-kan*	This work
ITK3	MG1655 *nnr*::*frt-kan*	This work
ITK4	MG1655ΔP_orn_::GAATTC *nnr*::*frt-kan*	This work
ITK5	MG1655ΔP_glyV-X-Y_::GAATTC *nnr*::*frt-kan*	This work
SR21	MG1655 *frt*-rrnBT-U	This work
SR22	MG1655 rrnBT-D-*frt*	This work
SR18	MG1655 ΔP_queG_::*frt*	This work
SR33	SH022 *frt*-rrnBT-U	This work
SR34	SH022 ΔP_queG_::*frt*	This work
MIT35	MG1655 Δ*DARS1*::*cat*	Fujimitsu et al. (2009)
SR31	MIT35 *frt*-rrnBT-U	This work
SR32	MIT35 ΔP_queG_::*frt*	This work
MIT78	MG1655 Δ*DARS2*::*cat*	Fujimitsu et al. (2009)
SR33	MIT78 *frt*-rrnBT-U	This work
SR34	MIT78 ΔP_queG_::*frt*	This work
ITK23c	KYA018 *queG*::*frt-kan*	This work
ITK24c	KYA018 *datA*subIBS *queG*::*frt-kan*	This work
ITK39	ITK9 *datA*subIBS *queG*::*frt-kan*	This work
ITK8c	SH022 ΔT_glyV-X-Y_ *nnr*::*frt-kan*	This work
ITK16c	SH022 *datA*subDnaAbox2 *nnr*::*frt-kan*	This work
ITK40	ITK9 ΔT_glyV-X-Y_ *nnr*::*frt-kan*	This work
ITK13	ITK9 *datA*subDnaAbox2 *nnr*::*frt-kan*	This work

A *datA* fragment spanning P_orn_ to P_queG_ was amplified by PCR from MG1655 genomic DNA and primers datA_LI and datA_RI (see Table 2 for primer sequences), digested with AatII and HindIII-HF, and inserted into the pACYC177 to yield pITK1. pITK1-derivatives for construction of genomic mutants were generated using inverse PCR with the following primers (Table 2): ΔPorn_L and ΔPorn_R for pITK2 (ΔP_orn_), ΔPgly1_L and ΔPgly1_R for pITK3 (ΔP_glyV-X-Y_), delter_L and delteronly_R for pITK12 (ΔT_glyV-X-Y_), and subDnaAbox2_L and subDnaAbox2_R for pITK15 (*datA*subDnaAbox2). A *glyV-X-Y* fragment was amplified by PCR from MG1655 genomic DNA using primers datAess_L and datAess_R, digested with BglII and HindIII-HF, and inserted into the pKP1673 to yield pITK6. A *frt-kan* fragment was amplified by PCR using pTH5 and primers 40kan-T1T2-L and 40kan-T1T2-R, digested with EcoRI-HF, and ligated with a EcoRI-HF-digested vector fragment that was amplified by inverse PCR using pBAD18 and primers 18-T1T2-L and 18-T1T2-R to yield pSR14 carrying *frt-kan* instead of the *bla* gene.

**TABLE 2:** List of oligonucleotides used in this study.

Names	Sequences
RT-datAIBS_L	CAGAGTTATCCACAGCCTCAGG
RT-datAIBS_R	CAAGTGATCGACTCGACAAAAC
RT-rpoA_L	CCCAGAGTATGGCCAAAGCC
RT-rpoA_R	CTGTGACAGAGTTCTAAAACCGC
ORI_1	CTGTGAATGATCGGTGATC
KWoriCRev	GTGGATAACTCTGTCAGGAAGCTTG
RT-orn1_L	GAGCGCGATCGCATTATTGA
RT-orn1_R	TGGTGTACTGCAATGGTCGG
RT-orn2_L	TGACCGATGCCAACCTGAAT
RT-orn2_R	CGCACGTTCCAGTCATCCAT
RT-orn3_L	TGAACTGGCAACGCTCGAAT
RT-orn3_R	CTTCCAGCTCCGGCATGTAT
RT-orn4s_L	GCCGGAGCTGGAAGCCTACTTC
RT-orn4s_R	GCTTGGTAAAACCATCCAGAATTTCC
RT-PglyVXY_U1_L	TCAGCACTTGAGATAAAAACGC
RT-PglyVXY_U1_R	ATTTCTGCGTCGTTACGGGA
RT-PglyVXY_U2_L	CGTATAATGCGCCTCCCGTA
RT-PglyVXY_U2_R	TCCCGCGTACTACTTAATTTTGC
RT-PglyVXY_D1_L	AACGACGCAGAAATGCGAAAA
RT-PglyVXY_D1_R	TCCCGCGTACTACTTAATTTTGCT
RT-PglyVXY_D2_L	GTAACGACGCAGAAATGCGA
RT-PglyVXY_D2_R	CAACTGAGCTATTCCCGCGTA
pTH5-KanL1	GCAGAAATGCGAAAATTACGAAAGCAAAATTAAGTAGTACATGAGGATCGTTTCGCATGATTG
pTH5-KanR	CGCATCGCGTCGCTGTGGATATTTTATTGAGAGAAGAATTTCAGAAGAACTCGTCAAG
Kan1_L	GATGGATTGCACGCAGGTTC
Kan1_R	CAGCCGATTGTCTGTTGTGC
datAIBS-70-L	ACTCGACAAAACGGAATCTTAATTTTTAACTTATCTATCAATAGGTTAAAAAAATACTATTCACCGTGCG
Northern_leftIBS1	CCTGATAGCGGTCCGCCACACCCAGCCGGCC
rpoA-Theisen	GGCGCGGTTTTAGAAACTCTGTCACAGAACCCTG
pTH5-kan_L_f	TCGCGCTGGCGAGGCCGCATTCCAGGTGTGTCGTTCGGCGTATCCTGACGGCTGCTGGGATTACACATGGC
pTH5-kan_R	CGTAGCCATCGCCGCCGTTATTACCATGACCGCACAGCACCAGCCAGTGGGTGTAGGCTGGAGCTGCTTC
datA_LI	CAGAAGCTTCGAGGTACGCTCATGCACCGCTTCC
datA_RI	CAGGACGTCCGTCAGGATACGCCGAACGACACAC
ΔPgly1_L	CAGGAATTCCCCCCCAAAAAAGTTTTTTTTGC
ΔPgly1_R	CAGGAATTCGCGCCTCCCGTAACGACGCAGAA
ΔPorn_L	CAGGAATTCAGATGTTTTGCCCATCAGGGGCG
ΔPorn_R	CAGGAATTCCACTGTGAATGGGTGGAAAATAG
datAess_L	CAGAGATCTCTCCAGGCCATTGTTTTGTCG
datAess_R	CAGAAGCTTCTGAGGCTGTGGATAACTCTG
40kan-T1T2-L	CGCGCGATATCAGAGAATTCCATATGAATATCCTCCTTAG
40kan-T1T2-R	CGCGCGATATCCAATAAAATATCCACAGCGACGC
18T1T2-L	CGCGCGATATCTGCCTGGCGGCAGTAGCG
18T1T2-R	CGCGGGATATCAAGGCCCAGTCTTTCGACTGAGC
T1T2-frtkan-L	GGGTCGCGAGTTCGAGTCTCGTTTCCCGCTCCAAATTCTTCTCTGATTAGCGGATCCTACCTGACGC
T1T2-frtkan-R	ACCAGCAATAACGCATCGCGTCGCTGTGGATATTTTATTGCTTAATTTGATGCCTGGCAGTTTATGGC
T1T2queGP-L	CTAACTGATTGAGATCGAGGGGCTCTGACATGACGGACCATTAATTTGATGCCTGGCAGTTTATGGC
PqueG-U-3	GCATCAGCTCATAGAGTGTGAGCCCCAGCACATCTGCCGCCTCGCGTTCTGTGTAGGCTGGAGCTGCTTC
PgueG-del-L2	TGACGGACCATACAATGAAGAAAAACCCCGCGATATCCGCCGCGGATGAGCTCTACAAATAATGAATTCC
ChdatA-U	GTGTAGGCTGGAGCTGCTTCCCACTACCGTTATCTCGATGTCAGC
ChdatAsubIBS	GACTCGACAAAACGGAATCTTAATTTTTAACTTATCGTACTTTAGGTTAAAAAAATACTATTCACCGTG
delter_L	TTATTGAGAGAAGAATTTGGAGC
delteronly_R	GCTTTGCTGGTTTTTGTTGTCTCTGAC
subDnaAbox2_L	ACACATGTTCTCTGTTTACAAGAGTTTGTC
subDnaAbox2_R	GCCTCAGGCTGTAATCTTAATTTC
datAfusion_L2	ATGACGATACCTTCATCAGGCTCGC
datA_R_f	CGTCAGGATACGCCGAACGACACACCTGGAATGCGGCCTCGCCAGCGCGA

**FIGURE 3:**
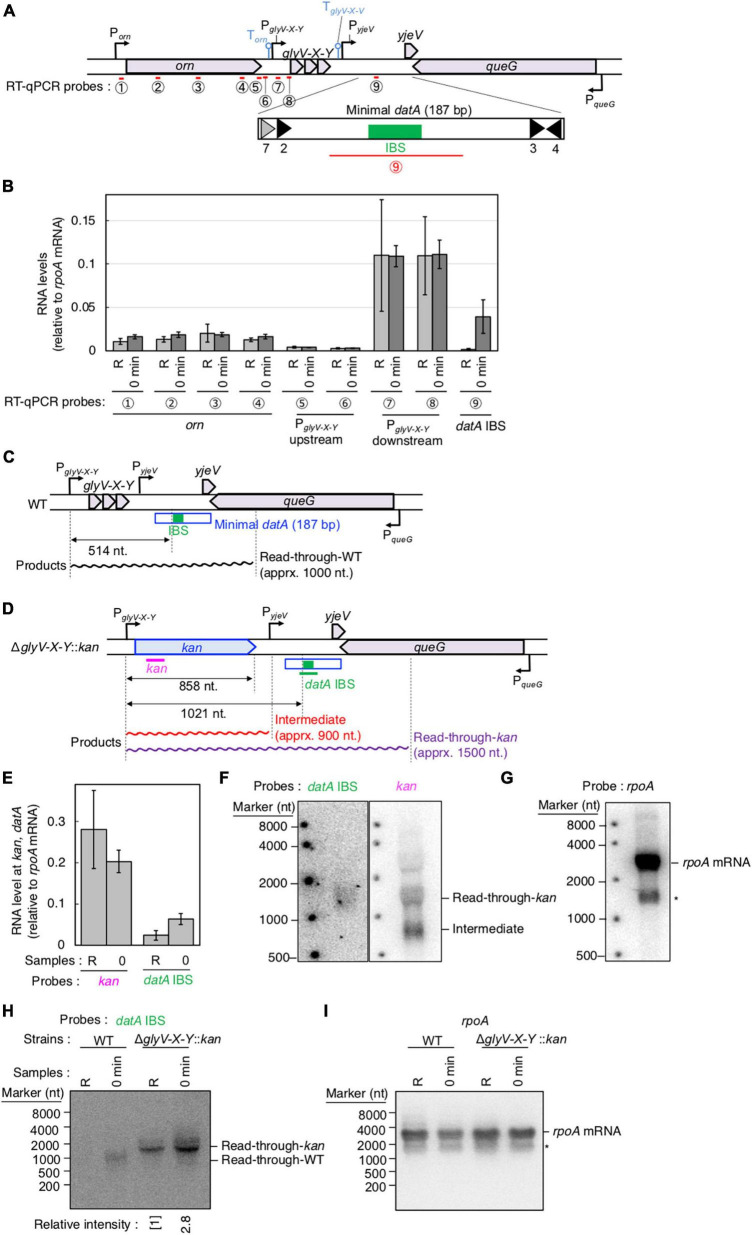
Determination of the transcriptional promoter for *datA* transcription. **(A)** Functional structures of *datA* and surrounding regions are shown as in Figure 2A. The amplified regions at *orn* gene, *glyV-X-Y* operon, and *datA* IBS locus are indicated by red bars: Each region is indicated by the encircled number. **(B)** SH022 cells growing in LB medium at 30°C (R; random) were transferred to 37°C and incubated for 80 min (0 min; synchronized), and then the total RNA was extracted. The RNA levels of each region relative to those of the *rpoA* gene were determined using RT-qPCR. Data represent means (± S.D.) of at least four independent experiments. The encircled numbers correspond to the regions shown in panel **(A)**. Based on the Student’s t-test, the R vs. 0 min difference (*p* < 0.007) using *datA* IBS probe is statistically significant. The effect of the *dnaC2*-dependent cell cycle synchronization was confirmed by checking the decrease of *mioC* transcript (Supplementary Figure 2A). **(C,D)** Construction of the Δ*glyV-X-Y*::*kan* mutant. The wild-type **(C)** and mutant **(D)** structures are illustrated. The probes at *kan* gene and *datA* IBS locus for northern blotting are shown as pink lines. The read-through *datA* RNA or its intermediate observed in northern blotting are shown as wave lines. **(E)** ITK9c (*dnaC2*Δ*glyV-X-Y*::*kan*) cells growing in LB medium at 30°C (R; random) were transferred to 37°C and incubated for 80 min (0 min; synchronized). The total RNAs were extracted at each time. The RNA levels of *kan* gene and *datA* IBS locus as well as *rpoA* gene were determined using RT-qPCR, and calculated as the relative value compared with those of the *rpoA* gene. Data represent means (± S.D.) of three independent experiments. Based on the Student’s t-test, the R vs. 0 min difference (*p* < 0.01) using *datA* IBS probe is statistically significant. The effect of the *dnaC2*-dependent cell cycle synchronization was confirmed by checking the decrease of *mioC* transcript (Supplementary Figure 2B). **(F,G)** Northern blotting using total RNA prepared from ITK9 (Δ*glyV-X-Y*::*kan*) cells and the probes for *datA* IBS [**(F)**; left], *kan* gene [**(F)**; right], and *rpoA* gene **(G)**. **(H,I)** Northern blotting using total RNA prepared from randomly-growing (R) or synchronized (0 min) *dnaC2* strains SH022 (WT) or ITK9c (Δ*glyV-X-Y*::*kan*). The probes for *datA* IBS **(H)** and *rpoA* gene **(I)** were used. The asterisk means unidentified bands.

**FIGURE 4:**
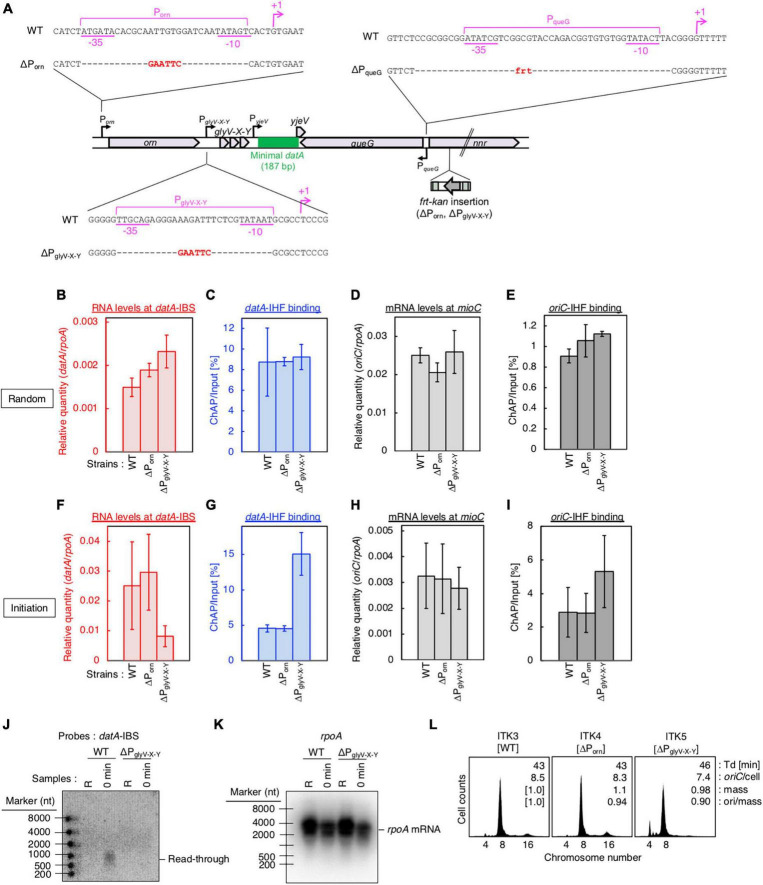
P_glyV-X-Y_ is required for transcription-dependent *datA* regulation. **(A)** Construction of transcriptional promoter mutant cells (ΔP_orn_ and ΔP_glyV-X-Y_ harboring an *frt-kan* insertion at the *nrr* gene, and ΔP_queG_::*frt*). **(B–I)** ITK3c (*dnaC2*) (WT), ITK4c (*dnaC2*ΔP_orn_) (ΔP_orn_), and ITK5c (*dnaC2*ΔP_glyV-X-Y_) (ΔP_glyV-X-Y_) cells growing in LB medium at 30°C [random culture; panels **(B–E)**] were transferred to 37°C and incubated for 80 min [synchronized at the initiation period; panels **(F–I)**]. The RNA levels of *datA* IBS **(B,F)** or the *mioC* gene **(D,H)** relative to those of the *rpoA* gene were determined using RT-qPCR. IHF binding at *datA* **(C,G)** and *oriC* **(D,I)** were determined by ChAP-qPCR, and yield was calculated (expressed as %). Data represent means (± S.D.) of at least two independent experiments. Based on the Student’s t-test, the WT vs. ΔP_glyV-X-Y_ differences in panel **(F)** (*p* < 0.12) and 4G (*p* < 0.01) are statistically significant. **(J,K)** Northern blotting using total RNA prepared from randomly-growing (R) or synchronized (0 min) ITK3c (WT) or ITK5c (ΔP_glyV-X-Y_) cells, and the probes for *datA* IBS **(J)** and *rpoA* gene **(K)**. **(L)** Flow cytometry analysis of ITK3 (WT), ITK4 (ΔP_orn_), and ITK5c (ΔP_glyV-X-Y_) cells. Cells were grown at 30°C in LB medium, followed by further incubation with rifampicin and cephalexin for run-out replication. DNA content was quantified using flow cytometry. Cell size (mass) at the time of drug addiction was measured using a Coulter counter. The *oriC*/cell, mean mass, and ori/mass ratio of each cell are indicated in each panel.

**FIGURE 5:**
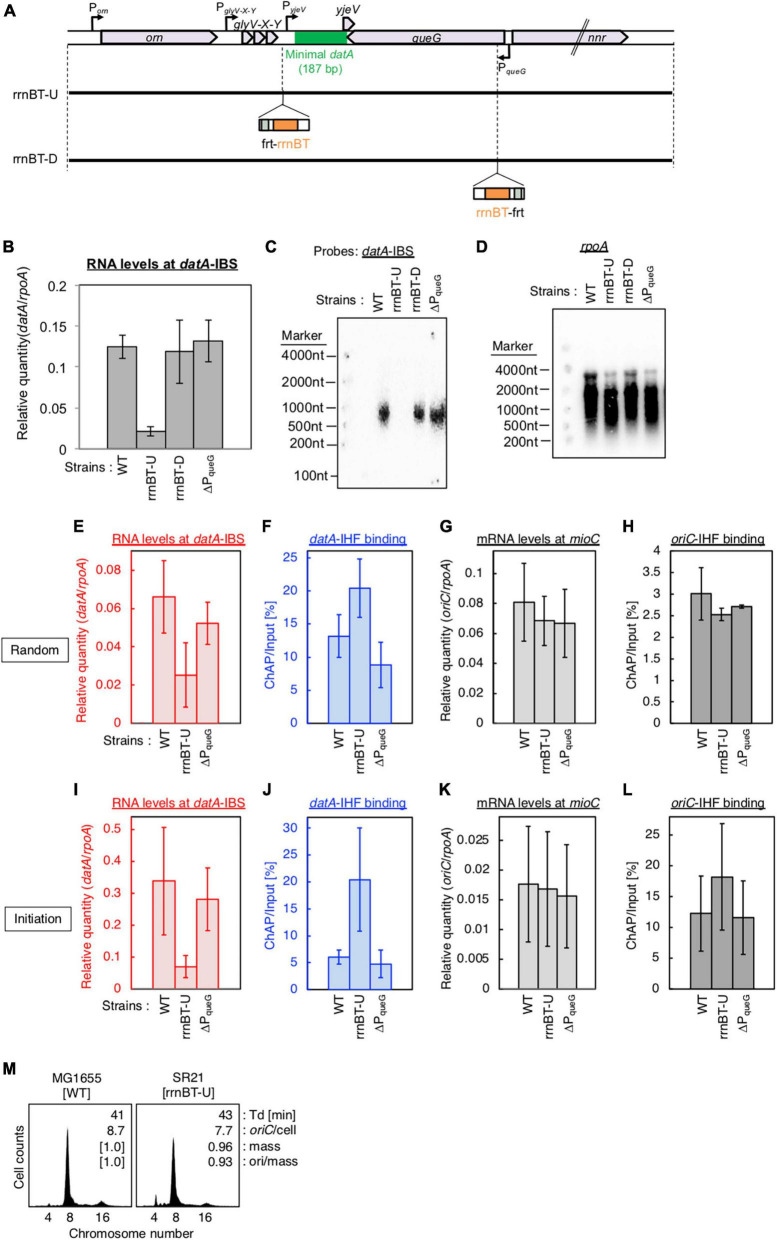
Insertion of a transcriptional terminator inhibits transcription-dependent *datA* regulation. **(A)** Construction of transcriptional terminator (rrnBT) insertion mutant strains. **(B)** MG1655 (WT), SR21 (rrnBT-U), SR22 (rrnBT-D), and SR18 (ΔP_queG_) were cultivated in LB medium at 37°C and then the total RNA was extracted. The RNA levels of *datA* IBS relative to those of the *rpoA* gene were determined using RT-qPCR. Data represent means with error bars of two independent experiments. Based on the Student’s t-test, the WT vs. rrnBT-U difference (*p* < 0.01) is statistically significant. **(C,D)** Northern blotting using total RNA prepared from MG1655, SR21, SR22, and SR18 cells and the probes for *datA* IBS **(C)** and *rpoA* gene **(D)**. **(E–L)** SH022 (*dnaC2 ihfA-cHis12*), SR35 (*dnaC2 ihfA-cHis12* rrnBT-U), and SR36 (*dnaC2 ihfA-cHis12* rrnBT-D) cells growing in LB medium at 30°C [random cultures; **(E–H)**] were transferred to 37°C and incubated for 80 min [synchronized; **(I–L)**]. The RNA levels of *datA* IBS **(E,I)** and *mioC* gene **(G,K)** relative to those of the *rpoA* gene were determined using RT-qPCR. IHF binding at *datA* **(F,J)** and *oriC* **(H,L)** were determined by ChAP-qPCR, and yield was calculated (expressed as %). Data represent means with error bars of two independent experiments. Based on the Student’s t-test, the WT vs. rrnBT-U differences in panel **(F)** (*p* < 0.04), 5I (*p* < 0.1) and 5J (*p* < 0.03) are statistically significant. **(M)** Flow cytometry analysis of rrnBT-U mutant cell. Cells were grown at 30°C in LB medium, followed by flow cytometry analysis.

**FIGURE 6:**
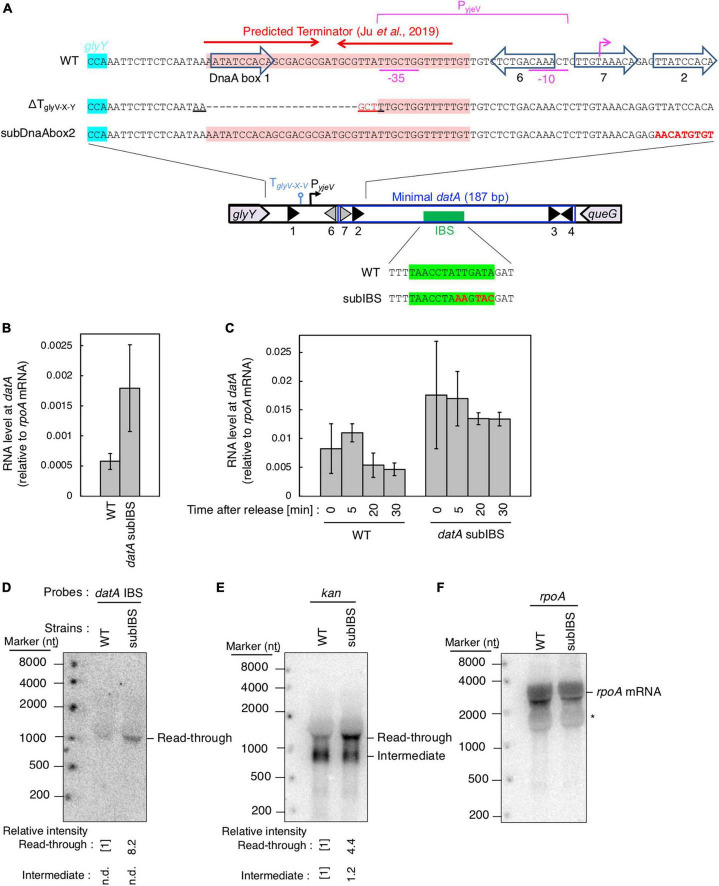
*datA*-IHF binding inhibits progression of *datA* transcription. **(A)** Construction of the mutant cells lacking transcriptional terminator/attenuator sequence (ΔT_glyV-X-Y_) and substitution mutant cells of *datA* DnaA box 2 or IBS (*datA*subDnaAbox2 or subIBS). **(B,C)** ITK23c (*dnaC2 queG::frt*) (WT) and ITK24c (ITK23c *datA*subIBS) (*datA*subIBS) cells growing in LB medium at 30°C [random cultures; **(B)**] were transferred to 37°C and incubated for 80 min [synchronized; **(C)**]. The cells were transferred to 30°C (Time 0) and further incubated for 5–15 min at 30°C, before total RNA was extracted. The RNA levels of *datA* IBS locus relative to those of the *rpoA* gene were determined using RT-qPCR. Data represent means (± S.D.) of at least three independent experiments. Based on the Student’s t-test, the WT vs. *datA* subIBS difference (*p* < 0.02) in panel **(B)** and 5 min vs. 20 min (*p* < 0.02) and 5 min vs. 30 min (*p* < 0.06) of the WT samples in panel **(C)** are statistically significant. The effect of the *dnaC2*-dependent cell cycle synchronization was confirmed by checking the decrease of *mioC* transcript (Supplementary Figure 2C). **(D–F)** Northern blotting using total RNA prepared from ITK9 (Δ*glyV-X-Y*::*kan datA* WT) and ITK39 (Δ*glyV-X-Y*::*kan datA*subIBS) cells, and the probes for *datA* IBS **(D)**, *kan* gene **(E)**, and *rpoA* gene **(F)**. The asterisk means unidentified bands.

For the construction of mutant strains in Figures 3, 5, 6, *datA* fragments carrying the desired mutation were amplified from mutant plasmids (pITK2 for ΔP_orn_, pITK3 for ΔP_glyV-X-Y_, pITK12 for ΔT_glyV-X-Y_ and pITK15 for *datA* subDnaAbox2) with primers datAfusion_L2 and datA_R_f (Table 2). Using the overlap extension (OE)-PCR method (Hilgarth and Lanigan, 2020), these fragments were joined with a *frt-kan* fragment amplified from pTH5 using primers pTH5-kan_L_f and pTH5-kan_R. Fragments were transformed into MG1655 or SH022 cells bearing pKD46 by electroporation, and the *frt-kan* was removed by introduction of the pCP20 plasmid, yielding strains ITK4 (ΔP_orn_), ITK5 (ΔP_glyV-X-Y_), ITK8 (ΔT_glyV-X-Y_), ITK16 (*datA*subDnaAbox2), ITK4c (*dnaC2*ΔP_orn_), ITK5c (*dnaC2*ΔP_glyV-X-Y_), ITK8c (*dnaC2*ΔT_glyV-X-Y_), and ITK16c (*dnaC2 datA*subDnaAbox2). For generation of the ITK3 control strain, a *frt-kan* fragment amplified from the pTH5 plasmid with primers pTH5-kan_L_f and pTH5-kan_R was transformed into MG1655 cells bearing pKD46. For the construction of mutant strains in Figure 4, fragments carrying a transcription terminator sequence of the *rrnB* operon (rrnBT) were amplified from pSR14 using primers T1T2-frtkan-L and T1T2-frtkan-R for rrnBT-U, and T1T2-queGP-L and T1T2-frtkan-R for rrnBT-D, and were transformed into MG1655 or SH022 cells bearing pKD46 by electroporation. The *frt-kan* was removed by introduction of the pCP20 plasmid, yielding strains SR21 (rrnBT-U), SR22 (rrnBT-D), and SR33 (*dnaC2* rrnBT-U). A fragment carrying *frt-kan* was amplified from pTH5 with primers PqueG-U-3 and PgueG-del-L2, and was transformed into MG1655 or SH022 cells bearing pKD46 by electroporation. The *frt-kan* was removed by introduction of the pCP20 plasmid, yielding strains SR18 (ΔP_queG_) and SR34 (*dnaC2* ΔP_queG_). A fragment carrying the *datA*subIBS mutation and *frt-kan* was amplified from pKX40kan (Kasho et al., 2017) with primers ChdatA-U and ChdatAsubIBS, and transformed into MG1655 or SH022 cells bearing pKD46 using electroporation. The *frt-kan* was removed by introduction of the pCP20 plasmid, yielding strains ITK24 (*datA*subIBS) and ITK24c (*dnaC2 datA*subIBS), respectively. For checking the introduction of specific mutations in each strains constructed in this study, the sequences of chromosomal *datA* locus were confirmed by DNA sequencing.

### Chromatin affinity precipitation combined with quantitative PCR (ChAP-qPCR)

Chromatin affinity precipitation (ChAP, a modified version of ChIP assay) and qPCR experiments were performed according to a previously described method (Kasho et al., 2014, 2021; Inoue et al., 2016). In brief, SH022 (*dnaC2 ihfA-cHis12*) or its derivatives were grown in LB medium at 30°C, a permissive temperature, until A_660_ of 0.04 was reached, after which cells were incubated at 37°C, a restrictive temperature, for 80 min (“0 min” sample). Cells were then incubated at 30°C for 5 min to initiate replication (“5 min” sample), followed by further incubation at 38°C for 5–25 min to allow a single round replication to proceed (“10–30 min” samples). For preparing random culture (“R”) samples, SH022 cells or their derivatives were grown in LB medium at 30°C until the A_660_ reached 0.15. Samples were cross-linked with 3% (final) formaldehyde at room temperature for 5 min. The reactions were quenched by incubation in 125 mM glycine for an additional 5 min. Then, the cells were collected by centrifugation, washed twice with 1 mL of ice-cold TBS [50 mM Tris–HCl (pH7.5) and 500 mM NaCl], resuspended in 500 μL of binding buffer [50 mM Tris–HCl (pH 7.5), 500 mM NaCl, 1% (vol/vol) Triton X-100, 5 mM imidazole, and 0.1 mM PMSF], and sonicated six times for 20 s each. The resulting size of the chromosomal DNA was about 1 kb. Cell debris was then removed by centrifugation at 16,000 × *g* for 15 min at 4 °C, and a portion (400 μL) of the resulting supernatant (450 μL) was mixed with 10 μL of Dynabeads His-tag Isolation and Pulldown (lifetechnologies), followed by incubation at 4°C for 1 h with a gentle rotation. Beads and bound materials were washed four times with wash buffer [50 mM Tris–HCl (pH 7.5), 500 mM NaCl, 1% (vol/vol) Triton X-100, and 5 mM imidazole], resuspended in elution buffer [50 mM Tris–HCl (pH 7.5), 500 mM imidazole, 1% SDS, 10 mM EDTA, and 10 mM dithiothreitol], and incubated at 65°C for 12 h to allow de-crosslinking. DNA in the samples before (Input) and after (ChAP) pull down was purified using a Wizard SV Gel and PCR Clean-Up System (Promega). The levels of *oriC*, *datA*, and *ylcC* were quantified by real-time qPCR using TB Green Premix Ex TaqII (Tli RNaseH Plus) (TaKaRa) and primers ORI_1 and KWoriCRev for *oriC*; RT-datAIBS-L and RT-datAIBS-R for *datA*; and RTYLCC-L and RTYLCC-R for *ylcC*.

### Total RNA purification

**FIGURE 7:**
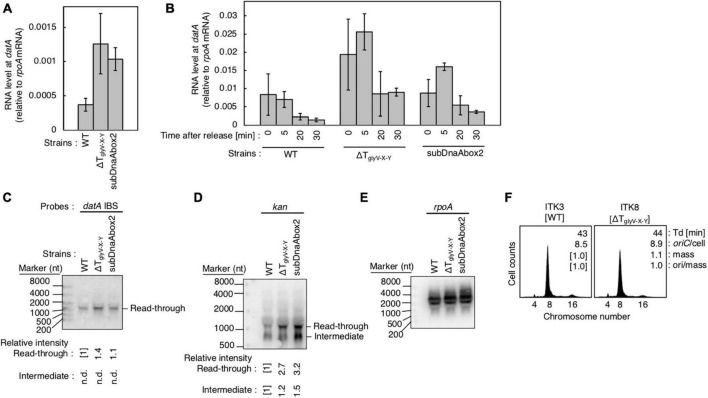
Analysis of transcriptional terminator/attenuator sequence of the *glyV-X-Y* operon and *datA* DnaA box 2 on transcription-dependent *datA* regulation. **(A,B)** ITK3c (*dnaC2*) (WT), ITK8c (*dnaC2*ΔT_glyV-X-Y_) (ΔT_glyV-X-Y_), and ITK16c (*dnaC2 datA*subDnaAbox2) (subDnaAbox2) cells growing in LB medium at 30°C (random cultures; A) were transferred to 37°C and incubated for 80 min **(B)**. The cells were transferred to 30°C (Time 0) and further incubated for 5–30 min at 30°C, and then the total RNA were extracted. The RNA levels of *datA* IBS locus relative to those of the *rpoA* gene were determined using RT-qPCR. Data represent means with error bars of at least two independent experiments. Based on the Student’s t-test, the WT vs. ΔT_glyV-X-Y_ (*p* < 0.04) and WT vs. subDnaAbox2 difference (*p* < 0.006) in panel **(A)**, 5 min vs. 20 min (*p* < 0.02) and 5 min vs. 30 min (*p* < 0.007) of the WT samples, 5 min vs. 30 min (*p* < 0.09) of the ΔT_glyV-X-Y_ samples, and 5 min vs. 20 min (*p* < 0.07) and 5 min vs. 30 min (*p* < 0.008) of the subDnaAbox2 samples in panel **(B)** are statistically significant. The effect of the *dnaC2*-dependent cell cycle synchronization was confirmed by checking the decrease of *mioC* transcript (Supplementary Figure 2D). **(C–E)** Northern blotting using total RNA prepared from randomly-growing ITK9 (*glyV-X-Y*::*kan datA* WT), ITK40 (*glyV-X-Y*::*kan* ΔT_glyV-X-Y_), or ITK13 (*glyV-X-Y*::*kan datA*subDnaAbox2) cells and the probes for *datA* IBS **(C)**, *kan* gene **(D)**, and *rpoA* gene **(E)**. **(F)** Flow cytometry analysis of ΔT_glyV-X-Y_ mutant cells. Cells were grown at 30°C in LB medium, followed by flow cytometry analysis.

Total cellular RNA samples used in Figures 2B, 3B, 5, and Supplementary Figure 1 were purified using NucleoSpin™ RNA (Macherey-Nagel) according to the manufacturer’s protocol. Total cellular RNA samples used in Figures 3E–I, 4, 6, 7 were purified using the hot phenol method (Su’etsugu et al., 2003). In brief, KYA018 (*dnaC2*), SH022, or their derivative cells were grown in LB medium at 30°C until they reached A_660_ of 0.04, followed by further incubation at 37°C for 80 min. Cells were then incubated at 30°C for 5 min, followed by further incubation at 38°C for 5–25 min. Samples were withdrawn at each of the indicated time-points and immediately mixed with equal volume of Stop buffer (20 mM sodium acetate, pH 5.2, 2% 30 mM sodium acetate-saturated phenol, pH 5.0, 2 mM EDTA, and 75% ethanol in diethylpyrocarbonate-treated MilliQ) to inactivate cellular RNase activity. The cells were then collected by centrifugation and resuspended with 400 μL of Resuspension buffer (20 mM sodium acetate, pH 5.2, 2% sodium dodecyl sulfate in diethylpyrocarbonate-treated MilliQ). Total RNA was then extracted twice by treatment with 400 μL 30 mM sodium acetate-saturated phenol (pH 5.0) and three times with 400 μL phenol/chloroform/isoamyl alcohol (25:24:1), collected by ethanol precipitation, and suspended with 50 μL RNase free dH_2_O (TaKaRa). The concentration of purified total RNA samples was determined using a NanoDrop™ Lite (Thermo Scientific).

### RT (reverse transcription)-qPCR

For RT-qPCR, cDNA samples were prepared using 1 μg of each total RNA sample and PrimeScript™ RT reagent Kit with gDNA Eraser (Perfect Real Time) (TaKaRa). The RNA levels were quantified by real-time qPCR using TB Green Premix Ex Taq™II (Tli RNaseH Plus) (TaKaRa) and primers ORI_1 and KWoriCRev for *oriC*; RT-datAIBS_L and RT-datAIBS_R for *datA*; RTrpoA-L and RTrpoA-R for *rpoA*; and RTmioC-L and RTmioC-R for *mioC*.

### Northern blotting

Northern blotting experiments were performed according to a previously described method (Su’etsugu et al., 2003), with the following minor modifications. Each transcript in purified total RNA samples was separated by electrophoresis using 2% agarose/2.2 M formaldehyde gel in 1x MOPS running buffer, transferred to Hybond™ N+ membrane (Cytiva) using the capillary blotting method, dried at 80°C for 2 h, and then fixed by exposure to UV (120 mJ/cm^2^) for 5 min using FUNA-UV-Crosslinker FS-800 (Funakoshi). Oligonucleotide probes with complementary sequences to either the *datA* IBS locus or the *rpoA* gene were end-labeled with ^32^P, and this labeled probe was hybridized with RNA on the membrane in the presence of ULTRAhyb™ Ultrasensitive Hybridization Buffer (Invitrogen). The membrane was then washed with 2x SSC (300 mM sodium chloride, 30 mM sodium citrate), 2x SSC+0.1% sodium dodecyl sulfate, 0.2x SSC (30 mM sodium chloride, 3 mM sodium citrate) +0.1% sodium dodecyl sulfate, and 0.2x SSC, dried, and analyzed using the Typhoon™ 7500 imaging analyzer (Cytiva).

## Results

### The levels of *datA* IBS transcription and *datA*-IHF binding fluctuate during the cell cycle

The minimal region of *datA* required for full DDAH activity is located at the intergenic region downstream of the tRNA-Gly (*glyV-X-Y*) operon between the *yjeV* gene and *queG* gene (Figure 2A). Indeed, the *datA* IBS was transcribed in exponentially-growing MG1655 (WT) cells when cultivated at 37°C in LB or supplemented M9 medium (Supplementary Figure 1A). Thus, we hypothesized that *datA*-IHF binding/dissociation is regulated by transcription of the *datA* locus. This theory is in accordance with previous observations that rifampicin inhibits timely dissociation of *datA*-IHF complexes (Kasho and Katayama, 2013), and that *datA* activity is inhibited by the translocation of *datA* to a highly transcribed position (Frimodt-Møller et al., 2015).

To analyze the cell cycle-dependent correlation between *datA*-IHF binding and transcription of the *datA* locus, we combined RT-qPCR and ChAP assays using temperature sensitive *dnaC2* cells, which allow synchronization of the cell cycle in LB medium (Withers and Bernander, 1998). DnaC is the helicase loader, and the *dnaC2* mutation inhibits replication initiation at *oriC* specifically at restrictive temperatures (37–42°C). Thus, the cell cycle of the *dnaC2* cells can be synchronized by incubation at 37°C for 80 min just before replication initiation (0 min), followed by a temperature down shift to 30°C for 5 min to release the cell cycle by activating DnaC, and induce a single round of replication initiation. An upshift back to 37°C for 10–30 min allows DNA synthesis to proceed while inhibiting additional replication initiation. This *dnaC2*-based synchronization system has previously been used to identify the cell cycle-coordinated IHF binding/dissociation pattern at *oriC*, *datA*, and *DARS2* (Kasho and Katayama, 2013; Kasho et al., 2014), as well as the cell cycle-specific oscillation of expression levels of genes including *dnaA*, *mioC*, and *gidA* (Theisen et al., 1993; Su’etsugu et al., 2003); thus, this well-established method is suitable for the purpose of this study.

To validate our assay, initial control experiments were performed in which the cell cycle-coordinated binding/dissociation of IHF at the *datA* and *oriC* loci was confirmed using ChAP-qPCR. In accordance with previous studies (Kasho and Katayama, 2013), at the *oriC* locus IHF was stably bound prior to initiation (0 min) and immediately dissociated after initiation (5–10 min) (Supplementary Figure 1B), while at the *datA* locus, IHF was not bound before initiation (0 min) and became bound after initiation (15–20 min) (Figure 2B).

Next, under the same experimental conditions as in Figure 2B, we prepared total RNA samples and performed RT-qPCR using primers recognizing *datA* IBS, the *mioC* gene as a control for cell cycle synchronization, or the housekeeping gene *rpoA* gene as a loading control (Su’etsugu et al., 2003). Consistent with a previous report, the mRNA level of the *mioC* gene relative to that of *rpoA* was lower at 0 min (Supplementary Figure 1D), indicating that the *dnaC2* cells were properly synchronized before initiation. By contrast, the relative RNA level at *datA* IBS was very low in the non-synchronized cells, dramatically increased at the initiation period (0–5 min), and decreased after initiation (10–20 min) (Figure 2B; Supplementary Figure 1C). No dramatic increase in the *datA* RNA level or decrease in the *mioC* mRNA level was observed when MG1655 (*dnaC* WT) cells were incubated at 37°C (Supplementary Figures 1C, D), indicating that transcription of *datA* is regulated in a cell cycle-dependent manner. A similar oscillation pattern of *datA* RNA level was observed using supplemented M9 medium (Supplementary Figure 1E). Thus, these results indicated that transcription of *datA* is regulated in a cell cycle-dependent manner.

In *dnaC2* cells, the level of *datA* RNA was approximately 8–40-fold higher at initiation (0 min) than in the random culture (R) sample (Figure 2B; Supplementary Figure 1C). The *datA* RNA levels were still higher at the post-initiation period in *dnaC2* cells than in random culture samples, which can also be explained by the accumulation of *datA* RNA as a result of cell cycle synchronization. Furthermore, the accumulation of *datA* RNA might affect the overall high baseline *datA* RNA level after releasing the cell cycle, i.e., during 5–10 min incubation, it is possible that *datA* RNA is not significantly degraded but is also newly produced. Thus, we concluded that the levels of IHF binding and transcription at the *datA* locus are inversely correlated with each other, supporting the idea that the *datA*-IHF binding/dissociation is regulated in a manner dependent on transcription.

### *glyV-X-Y* promoter is predominant for read-through transcription covering *datA*-IHF

To determine the transcriptional start site of *datA* RNA, we performed RT-qPCR using four primer sets for the orn gene, two sets each for the region upstream or downstream of the transcriptional promoter for *glyV-X-Y* operon (P_glyV-X-Y_), and one for *datA* IBS (Figure 3A). While the RNA levels of the orn gene and the *glyV-X-Y* transcription units remained constant, the relative RNA level of *datA* IBS fluctuated during the cell cycle and significantly increased at initiation, consistent with Supplementary Figure 1C (Figure 3B; probe 9). The relative mRNA levels of orn gene (probes 1–4) and the region upstream of the transcriptional promoter P_glyV-X-Y_ (probes 5 and 6) were much lower than those of the *glyV-X-Y* operon (probes 7 and 8) or *datA* IBS (probe 9), suggesting that transcription of the *orn* gene is substantially terminated at the P_glyV-X-Y_ upstream locus.

To confirm whether *datA* transcription is initiated at P_glyV-X-Y_, northern blotting was used to analyze the length of the *datA*-containing RNA products. The relative amounts of *datA*-IBS RNA were low (Figure 3B), which may result from degradation of the 3′-region of RNA products including tRNA-Gly during the tRNA maturation processes (Wellner et al., 2018). Thus, to efficiently analyze full-length RNA products that read through the *datA* IBS locus, we constructed the Δ*glyV-X-Y::kan* strain, in which the entire *glyV-X-Y* operon was replaced by the *kan* gene (Figures 3C, D). To avoid cell lethality resulting from lack of tRNA-Gly, tRNA-Gly was supplemented from the pITK6 plasmid-encoded *glyV-X-Y* in all the Δ*glyV-X-Y::kan* mutant cells constructed in this study.

In the ITK9c (*dnaC2* Δ*glyV-X-Y::kan*) cells, the level of *datA* RNA was significantly lower than that of the *kan* gene (Figures 3B, E; “R” sample), suggesting that transcription inhibition occurs at the *datA*-upstream region. Furthermore, the *datA* RNA level was 3-fold higher at initiation compared with randomly-growing cells (Figure 3E; “0 min” sample). Consistent with the RT-qPCR experiment, the northern blotting experiments revealed that the read-through RNA products, including *datA* IBS (approximately 1,500 nt), were observed using either the *datA* IBS probe or the *kan* probe (Figures 3F, G) and were increased at the initiation period (Figures 3H, I). Notably, the shorter RNA intermediate (approximately 800 nt) was only observed using the *kan* probe (Figure 3F). Given the observed fluctuation of *datA*-IBS RNA, these data suggest that the transcripts initiated from P_glyV-X-Y_ pass through *datA* IBS specifically at the initiation period and are terminated in front of *datA* IBS after initiation. We thus hypothesized that the specific DNA element located between the region downstream of *glyV-X-Y* and *datA* IBS is required for the cell cycle-coordinated *datA*-IBS transcription. Consistently, the relative RNA levels initiated from P_glyV-X-Y_ were not increased at the initiation period in either WT or Δ*glyV-X-Y::kan* cells (Figures 3B, E), supporting our hypothesis. The relative *datA*-IBS transcription levels in Figure 3B were moderately lower than those in Figure 2B, which might be due to subtle differences in cell-cultivation conditions affecting rates of transcription and processing of the full-length *datA* RNA products, including tRNA-Gly.

### Transcription-dependent regulation for *datA*-IHF binding

Next, to address the requirement of P_glyV-X-Y_ for transcription-dependent regulation of *datA*-IHF binding, we constructed ITK4c (*dnaC2* ΔP_orn_) and ITK5c (*dnaC2* ΔP_glyV-X-Y_) strains by inserting *frt-kan* into the *nnr* gene located close to, but outside of, the *datA* region (Figure 4A). In random cultures (Figures 4B–E), the *nnr::frt-kan* mutation had little effect on cell cycle-coordinated *datA*-IHF binding and *datA* transcription (Figures 4B, C), or *oriC*-IHF binding and *mioC* mRNA level (Figures 4D, E). We thus used ITK3c (*dnaC2 nnr::frt-kan*) cells as a WT control for analyzing cell cycle-coordinated oscillation of *datA* RNA and *datA*-IHF binding/dissociation.

In cells synchronized at the initiation period (Figures 4F, G), introduction of the ΔP_glyV-X-Y_ mutation greatly decreased the *datA*-IBS RNA level compared with that of ΔP_orn_ and the WT control and, notably, diminished the initiation period-specific decrease in *datA*-IHF binding (Figures 4F, G). Low residual RNA levels could be due to read-through RNA products from P_orn_. In contrast to *datA* IBS RNA, the *mioC* mRNA level was not substantially changed by introduction of the ΔP_glyV-X-Y_ mutation (Figure 4H). There may be a minor increase in the *oriC*-IHF binding level in ΔP_glyV-X-Y_ mutant cells, which could be an indirect consequence of a moderate delay in replication initiation induced by increased *datA*-IHF binding (see below). Consistent with qPCR analysis, northern blotting supports the requirement of P_glyV-X-Y_ for the initiation period-specific production of read-through *datA* RNA (Figures 4J, K).

To address the impact of the ΔP_glyV-X-Y_ mutation on regulation of replication initiation, we performed flow cytometry analysis. Cells were exponentially grown in LB medium and further incubated in the presence of rifampicin and cephalexin, which inhibit replication initiation and cell division, respectively, to complete replication. Cell mass (cell size) and chromosomal DNA content were then analyzed by flow cytometry to allow determination of the *oriC*/cell and the ori/mass, and thus the frequency of replication initiation. While WT cells predominantly showed peaks corresponding to four and eight origins, ΔP_glyV-X-Y_, but not ΔP_orn_, cells showed a significant increase in the peak corresponding to four origins and a decrease in the eight origins peak (Figure 4L). This means that the ΔP_glyV-X-Y_ mutation specifically decreased initiation frequency (ori/cell or ori/mass) (Figure 4L), consistent with our observation that P_glyV-X-Y_ is required for production of *datA* RNA and transcription-dependent regulation of *datA*-IHF binding. Given that IHF dissociates from *oriC* immediately after initiation, delay of initiation could result in an increased level of *oriC*-IHF complexes (Figure 4I).

To confirm the requirement of P_glyV-X-Y_ for regulation of *datA* function, we inserted the *frt* site-flanking strong transcriptional terminator element (rrnBT; terminator of *E. coli rrnB* operon) (Orosz et al., 1991) either upstream or downstream of *datA* IBS (rrnBT-U or -D, respectively) using the inserting *frt* site (Figure 5A). The rrnBT-U cells, but not the rrnBT-D or ΔP_queG_ cells, had significantly decreased levels of *datA*-IBS RNA compared with WT cells (Figure 5B). Consistent results were obtained by Northern blotting where the read-through RNA products including *datA* IBS (500–1,000 nt) were observed in WT, rrnBT-D, and ΔP_queG_ cells, but not in rrnBT-U cells (Figures 5C, D). Importantly, insertion of rrnBT-U specifically impaired timely *datA*-IHF dissociation and *datA* transcription at the initiation period, while stable *oriC*-IHF binding was maintained in rrnBT-U cells (Figures 5E–L). An observed slight increase in levels of *oriC*-IHF binding in rrnBT-U cells could be due to delayed initiation as for ΔP_glyV-X-Y_ (Figure 5L). Consistently, rrnBT-U cells showed moderately delayed initiation frequency in WT, Δ*DARS1*, or Δ*DARS2* backgrounds, whereas ΔP_queG_ cells did not (Figure 5G; Supplementary Figure 3). Also, in the rrnBT-U cells, rrnBT was inserted at the region upstream of T_glyV-X-Y_ to avoid affecting P_yjeV_ transcription, thus suggesting that P_glyV-X-Y_ has a predominant role in transcription-dependent *datA*-IHF regulation and the requirement of P_yjeV_ is limited. Taken together, these observations support the idea that P_glyV-X-Y_ is critical for the transcriptional regulation of timely *datA*-IHF binding.

### *datA*-proximal DNA elements are required for the timely oscillation of *datA* transcription

To address the regulatory mechanism and the requirement of the specific DNA element located between the region downstream of the *glyV-X-Y* operon and the *datA* IBS for transcription-dependent *datA* regulation, we used substitution mutants at *datA* DnaA box2 and IBS (subDnaAbox2 and subIBS, respectively) and a deletion mutant in which a predicted terminator/attenuator sequence T_glyV-X-Y_ of the *glyV-X-Y* operon was removed (ΔT_glyV-X-Y_) (Figure 6A; Ju et al., 2019). Because a previous study suggested that IHF binding at the promoter-attenuator region of the *ilvG-M-E-D-A* operon induces the pausing of transcription machinery (Pagel and Hatfield, 1991), we first tested whether *datA*-IHF binding interrupts transcription. For this purpose, we analyzed transcription levels of the *datA* IBS region in *dnaC2* or *dnaC2 datA*subIBS double mutant cells using RT-qPCR and primer set No. 9 from Figure 3A because these primers hybridize to sites outside of the *datA* IBS.

In *dnaC2 datA* WT cells, *datA* RNA was abundant during the initiation period (0–5 min) and decreased after initiation (20–30 min) (Figures 6B, C). By contrast, introduction of the *datA*subIBS mutation diminished this oscillation and the level of *datA* RNA remained constant. The constant increase of *datA* RNA throughout cell cycle resulted in accumulation of transcript and, therefore, an overall increase in *datA* RNA levels relative to WT cells in random culture samples (Figure 6B). Consistent with Figures 6B, C, introduction of the *datA*subIBS mutation into *dnaC2* Δ*glyV-X-Y::kan* cells resulted in increased total *datA* RNA levels (Figure 6D), and notably also increased the level of read-through *datA* RNA (*datA* IBS or *kan* probes; approximately 1,500 nt) relative to that of the RNA intermediate (*kan* probe; approximately 900 nt) (Figures 6D–F). This suggests that *datA*-IHF binding at the post-initiation period is stable enough to significantly interrupt transcription. However, a slight increase in the *datA* RNA level at the initiation period was observed, even in the *dnaC2 datA*subIBS cells (Figures 6B, C), implying that other potential regulatory elements may exist to interrupt *datA* transcription.

To address the possible role of T_glyV-X-Y_ for regulating transcription of the *datA* locus, we constructed a deletion mutant of T_glyV-X-Y_ (ΔT_glyV-X-Y_; Figure 6A) (Ju et al., 2019). T_glyV-X-Y_ overlaps with *datA* DnaA box 1 and the −35 sequence of a putative promoter, P_yjeV_ (Kitagawa et al., 1996). Therefore, we chose to delete *datA* DnaA box 1, which is dispensable for DDAH activity (Kasho and Katayama, 2013; Kasho et al., 2017), but not P_yjeV_. In addition, because previous studies suggested that DnaA binding around the terminator sequence of the *asnC* gene inhibits the passage of transcription machinery (Schaefer and Messer, 1988, 1989), we also addressed the possibility that the DnaA binding at the upstream region of *datA* IBS can act as a roadblock for transcription from P_glyV-X-Y_. Since *datA* DnaA box 2 is essential for ATP-DnaA-specific oligomer formation at *datA*, we constructed a *datA* mutant with DnaA box 2 substitution (*datA*subDnaAbox2) (Figure 6A), which lacks the DDAH activity of *datA* (Kasho and Katayama, 2013; Kasho et al., 2017).

Introduction of ΔT_glyV-X-Y_ or the *datA*subDnaAbox2 mutation resulted in increased levels of *datA*-IBS RNA at the initiation period (0–5 min) (Figures 7A, B). In contrast to the *datA*subIBS mutant cells (Figures 6B, C), both the ΔT_glyV-X-Y_ and *datA*subDnaAbox2 mutant cells sustained cell cycle-coordinated oscillation of *datA* RNA levels, although *datA* RNA levels at the post-initiation period (20–30 min) were also increased compared with those of WT cells. This suggests that either T_glyV-X-Y_ or *datA* DnaA box 2 may assist in regulation of the overall transcription level of *datA* but are not essential for the timely dissociation of the *datA*-IHF complex. Consistently, northern blotting analysis of *datA*-containing transcripts showed that the level of read-through *datA* RNA (IBS or *kan* probes; approximately 1,500 nt) relative to the intermediate (*kan* probe; approximately 900 nt) was only moderately increased in ΔT_glyV-X-Y_ and *datA*subDnaAbox2 cells compared with WT cells (Figures 7C–E). Despite the increase in basal *datA* transcription levels, the ΔT_glyV-X-Y_ mutation had little effect on initiation timing (Figure 7F). These results suggest that transcription block by T_glyV-X-Y_ or the DnaA binding at *datA* DnaA boxes 2 play specific roles in regulating the basal level of *datA* transcription but is dispensable for its cell cycle-coordinated oscillation and for controlling the timing of initiation. Taken together, these results suggest that another regulatory factor may play a role in timely *datA*-IHF binding in a manner independent of *datA* transcription.

## Discussion

**FIGURE 8:**
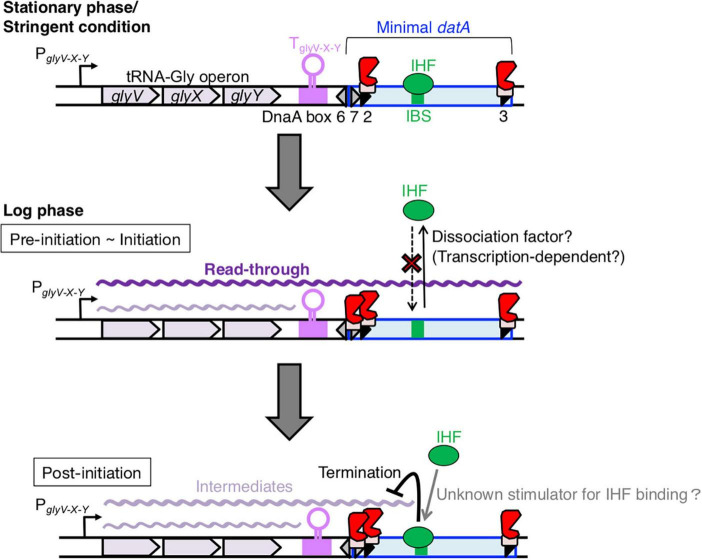
Model of tRNA transcription-dependent regulation for *datA*-IHF binding and dissociation during the cell cycle. In stationary phase cells, transcription of the tRNA-Gly (*glyV-X-Y*) operon is repressed (th*e* **top** panel). In the pre-initiation and initiation periods of growing cells (th*e* **middle** panel), the read-through transcription of the whole *datA* region is initiated from P_glyV-X-Y_ of the tRNA-Gly operon and is prerequisite for inhibiting *datA*-IHF binding or stimulating *datA*-IHF dissociation. An unknown factor might participate in this inhibition. The level of read-through *datA* transcription is constantly down-regulated by DnaA binding at *datA* DnaA box 2 and by the transcription terminator/attenuator sequence T_glyV-X-Y_ of the tRNA-Gly operon. In the post-initiation period (the **bottom** panel), IHF binding to *datA* IBS is stimulated/stabilized by an unknown mechanism and strongly inhibits read-through *datA* transcription at the site, resulting in timely activation of DDAH for repressing excess initiations.

Chromosomal DNA replication in bacteria is initiated at the origin of replication *oriC* and its timing is tightly regulated to ensure that firing at the replication origin occurs only once per cell cycle. The timely initiation is achieved through the activities of the replication initiator ATP-DnaA, whose cellular levels peak prior to initiation. And immediately after initiation, DnaA-bound ATP is hydrolyzed by RIDA and DDAH depending on *datA*-IHF complex to produce ADP-DnaA. To further understand how the timely IHF binding/dissociation at the *datA* locus is regulated, we investigated the requirement for transcription of the neighboring tRNA-Gly (*glyV-X-Y*) operon. Cell cycle analysis revealed that *datA*-IHF binding and read-through transcription of *datA* IBS are highly correlated during the cell cycle, i.e., IHF is not associated with *datA* at the initiation period and temporarily binds after initiation, while transcription of *datA* IBS is significantly decreased after initiation (Figure 2B). The timing of *datA*-IHF binding largely corresponds to that of *datA* duplication (Kasho and Katayama, 2013), implying the possibility that the regulation of *datA*-IHF binding is related to the progression of replication forks. Deletion of P_glyV-X-Y_ or insertion of a transcriptional terminator at the region downstream of glyY gene resulted in inhibition of *datA* transcription and enhanced *datA*-IHF binding (Figures 4, 5), indicating that transcription derived from P_glyV-X-Y_ and read-through transcription of *datA* IBS is required for timely dissociation of the *datA*-IHF complex (Figure 8). These mutations moderately inhibit replication initiation *in vivo* (Figures 4L, 5M), supporting the idea that P_glyV-X-Y_-derived transcription is required for down-regulation of *datA* function by reducing the chance of *datA*-IHF binding and that the chromosomal locus spanning tRNA-Gly operon to *datA* could function as a connecting hub for balancing the tRNA transcription level and the timing of replication initiation by sensing the cellular growth environment or stresses. Transcription of *E. coli* tRNA is globally regulated by Fis in a growth phase-dependent manner, and peaks at the exponential phase (Bosch et al., 1990; Nilsson et al., 1992). Other studies have suggested that some specific stringent conditions, such as starvation of isoleucine or folate, decrease the transcription level of most tRNAs (Ikemura and Dahlberg, 1973; Irving et al., 2021). A decrease in *datA* read-through transcription will stimulate constitutive *datA*-IHF binding, enhancing inactivation of DnaA. Taken together, we point to the possibility that *E. coli* cells fine-tune the DnaA activity through a regulatory mechanism that coordinates the tRNA-Gly transcription levels and concomitantly the basal amounts of read-through *datA* transcripts upon adaptation to various growing conditions such as exponential phase of cell growth or stringent condition.

Because the cell cycle analysis suggested that transcriptional initiation of the *glyV-X-Y* operon does not greatly increase in a replication initiation-specific manner (Figures 3A, B), we next addressed requirements of the specific DNA elements located between the region downstream of *glyY* and *datA* (IBS, DnaA box 2, and its proximal terminator/attenuator sequence T_glyV-X-Y_ of the *glyV-X-Y* operon) for the transcription termination. Importantly, in the *datA*subIBS mutant cells, the *datA* RNA level increased at the initiation period and did not substantially decrease during the ensuing 30 min after initiation, which is markedly different to WT *datA* cells (Figure 6C). Taken together, we suggest that the *datA*-IHF complex regulates cell cycle-coordinated attenuation of *datA* transcription and interrupts read-through *datA* transcription in a timely manner after initiation (Figure 8). This is consistent with previous studies showing the timely binding of IHF to *datA* and the transcription-termination activity of DNA-bound IHF (Pagel and Hatfield, 1991).

In *datA*subDnaAbox2 mutant cells, the level of *datA* RNA increased at the initiation period and decreased shortly after initiation, like that of the WT *datA* cells, and the overall level of *datA* transcription was increased compared with that of WT cells (Figures 7A, B). The role of *datA* DnaA box 2 for transcription termination could be consistent with previous observations that the DnaA oligomer formed at the specific region of *asnC* terminator sequence inhibits the passage of transcription machinery and causes transcription pausing (Schaefer and Messer, 1988, 1989). RNA polymerase might directly interact with DnaA (Flåtten et al., 2009). Another possible role of the *datA* DnaA box 2 could be as a riboswitch for transcription attenuation. A recent study suggested that DnaA binds to the specific RNA sequence rDnaA box and plays a regulatory role for transcription termination (Yao et al., 2023).

In addition, we addressed the role of T_glyV-X-Y_ which should be considered as an attenuator because considerable read-through is allowed (Figure 3F; Ju et al., 2019). In *E. coli*, the transcription units of each tRNA include a typical Rho-independent terminator sequence just 10–25 bp away from their tRNA-coding regions (Komine et al., 1990; Ju et al., 2019), and the unique feature of *glyV-X-Y* is that the DnaA box and the −35 sequence of P_yjeV_ partly overlap with the T_glyV-X-Y_ sequence (Kitagawa et al., 1996). Our results suggest that partial deletion of T_glyV-X-Y_ which also loses *datA* DnaA box 1, as well as *datA*subDnaAbox2 mutation, increased the overall level of read-through transcription of *datA*, but allowed a decrease after replicational initiation (Figure 7B). Despite the increase in basal *datA* transcription levels, the ΔT_glyV-X-Y_ mutation little affected the initiation timing (Figure 7F), suggesting that T_glyV-X-Y_ is also important for the overall transcriptional attenuation but not required for the timely attenuation of *datA* transcription.

Based on these observations, we propose a model whereby read-through *datA* transcription is required to down-regulate *datA*-IHF binding and is likely terminated by the timely assembly of the *datA*-IHF complex after replication initiation (Figure 8). In this model, *datA* transcription is only a prerequisite for IHF dissociation. *datA* transcription by itself is not sufficient for controlling the timing of *datA*-IHF binding, suggesting that an unknown regulatory factor is simultaneously required for the timely *datA*-IHF dissociation. As tRNA-Gly transcription initiation from P_glyV-X-Y_ and transcriptional attenuation by *datA* DnaA box 2 and T_glyV-X-Y_ are likely to operate constitutively during cell cycle, regulation of the timing of IHF dissociation would be due to the timely function of this unknown factor.

Previous studies have shown that the expression of most tRNA operons is regulated according to growth conditions, and that the level of tRNA-Gly (anticodon: GCC) derived from the *glyV-X-Y* operon is higher in richer medium (Emilsson et al., 1993). In this study, the transcription levels of *datA* IBS in exponentially-growing cells were broadly similar under different growth conditions (LB or supplemented M9 medium; Supplementary Figure 1A), yet stringent conditions would decline tRNA transcription initiation from P_glyV-X-Y_. Therefore, the transcription attenuation might modulate the transcription level of *datA* IBS under different growth conditions. Since some bacterial species such as *Bacillus subtilis* and *Streptomyces coelicolor* also have DnaA box clusters analogous to *datA* for repressing untimely initiations (Smulczyk-Krawczyszyn et al., 2006; Okumura et al., 2012), our findings and further future studies may provide a universal concept for transcription-coupled regulation of initiator proteins.

## Data availability statement

The original contributions presented in this study are included in this article/Supplementary material, further inquiries can be directed to the corresponding author.

## Author contributions

KK: Conceptualization, Data curation, Funding acquisition, Investigation, Methodology, Project administration, Supervision, Visualization, Writing—original draft, Writing—review and editing. RyS: Conceptualization, Data curation, Investigation, Methodology, Visualization, Writing—review and editing. KI: Data curation, Investigation, Methodology, Visualization, Writing—review and editing. WN: Investigation, Writing—review and editing. RiS: Investigation, Writing—review and editing. TJ: Investigation, Writing—review and editing. SO: Supervision, Writing—review and editing. TK: Conceptualization, Funding acquisition, Project administration, Supervision, Visualization, Writing—review and editing.
