# Supplementary material for: Read-through transcription of tRNA underlies the cell cycle-dependent dissociation of IHF from the DnaA-inactivating sequence datA
Source: Front Microbiol. 2024 Feb 28;15:1360108. doi: 10.3389/fmicb.2024.1360108 (PMC10950094; doi:10.3389/fmicb.2024.1360108)
Supplement: Supplementary file 1 [file Data_Sheet_1.PDF]

# *Supplementary Material*

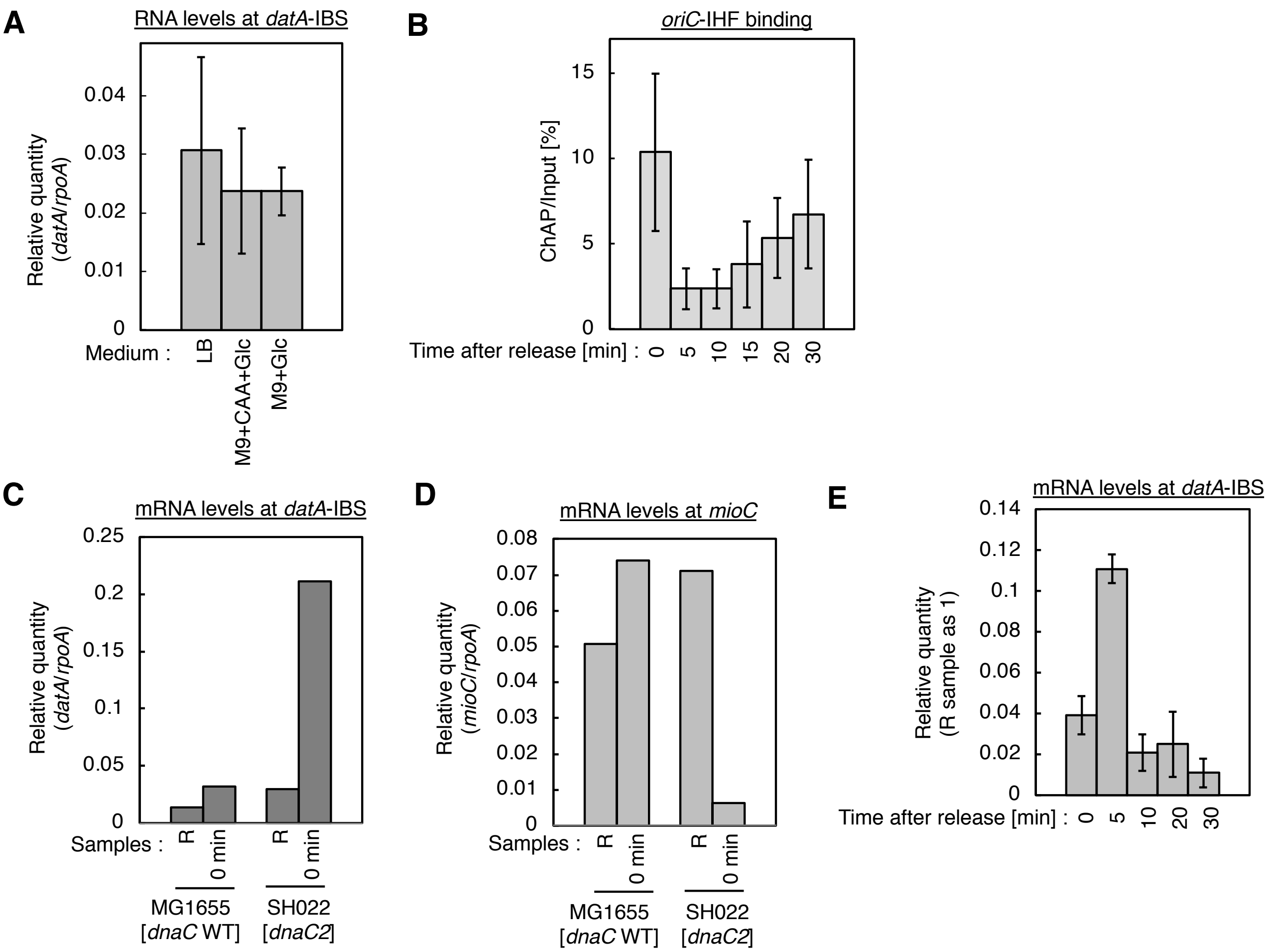


**SUPPLEMENTARY FIGURE S1.** Cell cycle-coordinated oscillation of *datA* transcription, related to Figure 1. (A) Detection of *datA*-containing RNA by RT-qPCR. MG1655 [WT] cells were cultivated in LB or supplemented M9 medium with or without 0.2% casamino acids at 37°C, and then the total RNA were extracted. The RNA levels *datA* IBS relative to those of the *rpoA* gene were determined using real-time qPCR. (B) Cell cycle-coordinated IHF binding/dissociation at *oriC*, analyzed by ChAP-qPCR. SH022 (*dnaC2 ihfA-cHis12*) cells growing in LB medium at 30°C were transferred to 37°C and incubated for 80 min. The cells were then transferred to 30°C (Time 0) and further incubated for 5–30 min at 30°C. The relative levels of *oriC* before and after Ni-affinity purification were determined using real-time qPCR, and yield was calculated (expressed as %). Data represent means (± S.D.) of four independent experiments. Cell cycle-coordinated oscillation of the RNA level of *datA* IBS (C) and of the *mioC* gene (D). MG1655 [WT] or SH022 [*dnaC2 ihfA-cHis12*] cells growing in LB medium at 30°C were transferred to 37°C and incubated for 80 min. The RNA levels of *datA* IBS and *mioC* gene relative to those of the *rpoA* gene were determined using real-time qPCR. (E) Cell cycle-coordinated

oscillation of the RNA level *datA* IBS in supplemented M9 medium. SH022 cells growing in supplemented M9 medium at 30°C were transferred to 38°C and incubated for 90 min. The RNA levels of *datA* IBS relative to those of the *rpoA* gene were determined using real-time qPCR. Data represent means with error bars of two independent experiments.
