# Supplementary material for: Read-through transcription of tRNA underlies the cell cycle-dependent dissociation of IHF from the DnaA-inactivating sequence datA
Source: Front Microbiol. 2024 Feb 28;15:1360108. doi: 10.3389/fmicb.2024.1360108 (PMC10950094; doi:10.3389/fmicb.2024.1360108)
Supplement: Supplementary file 2 [file Data_Sheet_2.PDF]

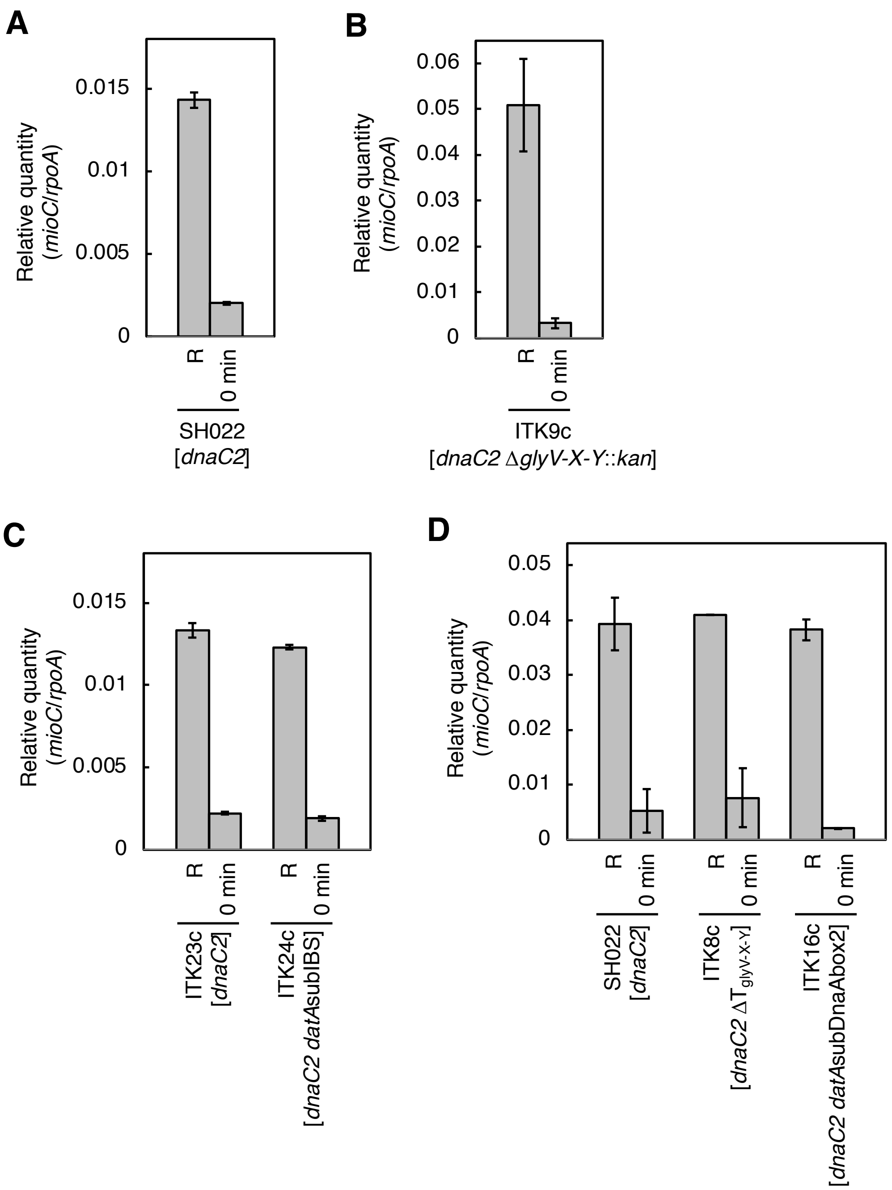

SUPPLEMENTARY FIGURE S2. Cell cycle-coordinated oscillation of *mioC* transcription, related to Figure 3, 6, and 7. Cell cycle-coordinated oscillation of *mioC* transcription was analyzed by RT-qPCR. *dnaC2* background cells SH022 (*dnaC2*) (A) related to Figure 3B, ITK9c (*dnaC2 ΔglyV-X-Y*::*kan*) (B) related to Figure 3E, ITK23c (*dnaC2*) and ITK24c (*dnaC2 datA*subIBS) (C) related to Figures 6B and C, and SH022 (*dnaC2*), ITK9c (*dnaC2* $\Delta T_{glyV\text{-}X\text{-}Y}$), and ITK16c (*dnaC2 datA*subDnaAbox2) (D) related to Figures 7A and B were grown in LB medium at 30°C (R; random culture samples), then transferred to 37°C, and further incubated for 80 min to synchronize the cell cycle (Time 0). The RNA levels of *mioC* gene relative to those of the *rpoA* gene were determined using real-time qPCR.
