# Supplementary material for: Read-through transcription of tRNA underlies the cell cycle-dependent dissociation of IHF from the DnaA-inactivating sequence datA
Source: Front Microbiol. 2024 Feb 28;15:1360108. doi: 10.3389/fmicb.2024.1360108 (PMC10950094; doi:10.3389/fmicb.2024.1360108)
Supplement: Supplementary file 3 [file Data_Sheet_3.PDF]

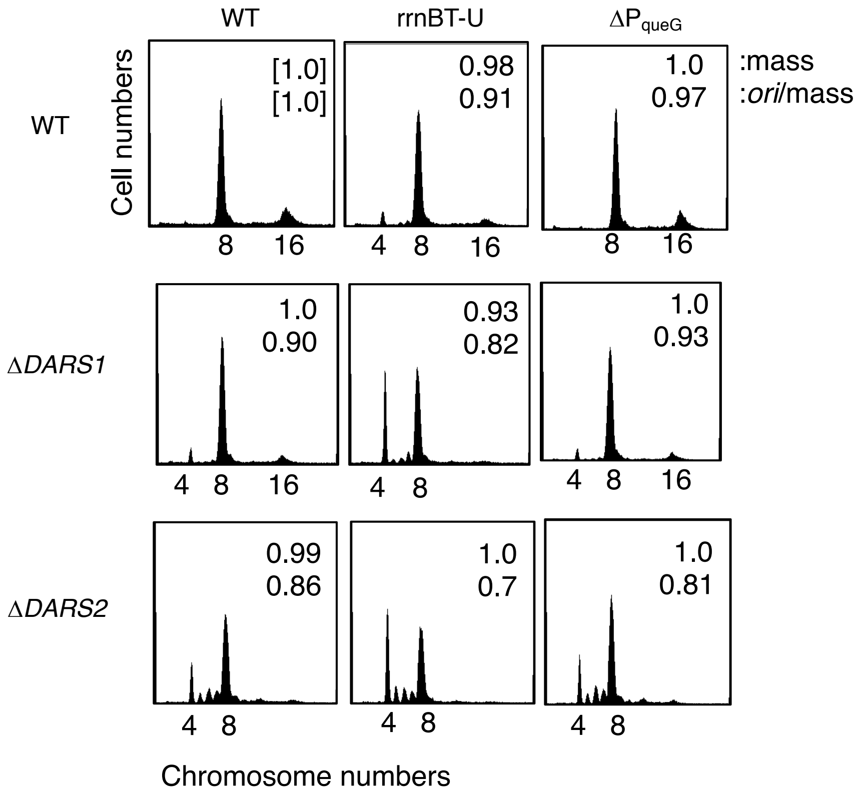

SUPPLEMENTARY FIGURE S3. Analysis of the effect of the transcriptional terminator/attenuator sequence of the *glyV-X-Y* operon on transcription-dependent *datA* regulation, related to FIGURE 5. Flow cytometry analysis of rrnBT-U or ΔP_{queG} mutant cells in Δ*DARS1/2* background. Cells were grown at 37°C in LB medium, followed by flow cytometry analysis.
